# Apps for asthma self-management: a systematic assessment of content and tools

**DOI:** 10.1186/1741-7015-10-144

**Published:** 2012-11-22

**Authors:** Kit Huckvale, Mate Car, Cecily Morrison, Josip Car

**Affiliations:** 1 Global eHealth Unit, Department of Primary Care and Public Health, Imperial College London, St Dunstan's Road, London W6 8RP, UK; 2 Engineering Design Centre, University of Cambridge, Trumpington Street, Cambridge CB2 1PZ, UK

**Keywords:** app, smartphone, mobile, ehealth, asthma, software, **TELEHEALTH**

## Abstract

**Background:**

Apps have been enthusiastically adopted by the general public. They are increasingly recognized by policy-makers as a potential medium for supporting self-management of long-term conditions. We assessed the degree to which current smartphone and tablet apps for people with asthma offer content and tools of appropriate quality to support asthma self-management.

**Methods:**

We adapted systematic review methodology to the assessment of apps. We identified English-language asthma apps for all ages through a systematic search of official app stores. We systematically assessed app content using criteria derived from international guidelines and systematic review of strategies for asthma self-management. We covered three domains: comprehensiveness of asthma information, consistency of advice with evidence and compliance with health information best practice principles.

**Results:**

We identified 103 apps for asthma in English, of which 56 were sources of information about the condition and 47 provided tools for the management of asthma. No apps offered both types of functionality. Only three information apps approached our definition of comprehensiveness of information about asthma. No apps provided advice on lay management of acute asthma that included details of appropriate reliever medication use. In 32 of 72 instances, apps made unequivocal recommendations about strategies for asthma control or prophylaxis that were unsupported by current evidence. Although 90% of apps stated a clear purpose, compliance with other best practice principles for health information was variable. Contact details were located for 55%, funding source for 18% and confidentiality policy for 17%.

**Conclusions:**

No apps for people with asthma combined reliable, comprehensive information about the condition with supportive tools for self-management. Healthcare professionals considering recommending apps to patients as part of asthma self-management should exercise caution, recognizing that some apps like calculators may be unsafe; that no current app will meet the need of every patient; and that ways of working must be adapted if apps are to be introduced, supported and sustained in routine care. Policy-makers need to consider the potential role for assurance mechanisms in relation to apps. There remains much to be done if apps are to find broad use in clinical practice; clinicians cannot recommend tools that are inaccurate, unsafe or lack an evidence base.

## Background

Apps, software specifically designed for and available on smartphones and tablets, have been enthusiastically adopted by users of smartphones and tablets and proposed as a delivery mechanism for self-management health interventions [1,2]. Forty-two percent of US adults have a phone with one or more apps and almost a third of these report having an app to help track or manage their health [3]. Policy-makers, concerned about growing demand associated with long-term conditions, think apps for patients might offer a scalable and convenient way to support the range of needs associated with self-management. Indeed, the UK Department of Health has suggested that apps be 'prescribed' as part of care for long-term conditions [4]. Proposals such as these motivate a question about whether current apps are suitable for this kind of use.

We address this by focussing on apps for asthma as a representative long-term condition. Asthma is common, globally-relevant, managed substantially in primary care and amenable to self-management. Moreover, while both the content of asthma self-management education has been well described in UK [5], US [6] and international [7] evidence-based guidelines, and its positive impact on outcomes demonstrated [8], the best way to communicate information and support its use through tools is less clear. Established methods such as face-to-face education and paper-based tools are inconsistently applied. For example, in the UK, only 20% of patients have ever received a written action plan [9]. Meanwhile, people with asthma are looking for alternatives; 65% report having used the internet to locate information about asthma without necessarily involving a health professional. Taken together, these create a specific opportunity for new methods to support self-management education.

This opportunity can only be realized if apps offer content and tools of appropriate quality. We adapted principles from systematic literature reviews to assess the content quality of smartphone and tablet apps for asthma against objective criteria derived from evidence-based guidelines [5]. We discuss our findings about asthma apps in relation to the wider policy context of prescribing apps to support the care and management of long-term conditions.

## Methods

### Selection of apps

**Figure 1:**
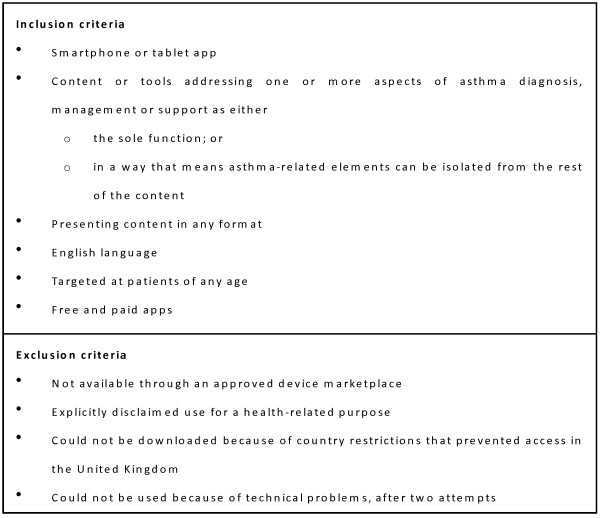
**Inclusion and exclusion criteria for smartphone apps**.

We aimed to identify all apps for asthma accessible to English-speaking patients. We searched the official app stores for Android, Apple, Blackberry and Windows Phone using the following terms: asthma, lung function, peak flow and inhaler. Apps were downloaded to test devices for screening by two authors (KH and MC) working independently using predefined inclusion and exclusion criteria (Figure 1). Test devices were unmodified consumer-grade smartphones running up-to-date versions of their mobile operating system. The same version of each app was used throughout testing.

### Planned assessment criteria

Basic details were extracted into a standard form (Additional file 1). Assessment was performed by two authors (KH and MC) assessing each app in a random order.

For apps presenting health information about asthma, we assessed two domains: the comprehensiveness of information about asthma and consistency of information with evidence-based guidelines on asthma. To assess comprehensiveness, we assessed coverage of eight topics recommended as the basis of self-management education and that are consistent across UK, US and international guidelines [5-7] (Table 1). For each topic, we assessed coverage as either 'complete', 'partial' or 'absent'. To evaluate consistency with evidence-based guidelines, we extracted a set of specific statements relating to secondary prevention and lifestyle advice from the same guidelines (Table 2). For each statement, we also captured the direction of the guideline recommendation indicating whether a particular strategy was 'beneficial', 'not beneficial' or whether there was insufficient or unclear evidence to be able to make a specific recommendation. To ensure fair assessment of apps sourced from multiple countries of origin, we retained only those statements considered by all three international guidelines used in the review. App content was reviewed and information corresponding to particular statements was assessed to see if any recommendation was consistent with that of the guidelines. Advice about actions for lay people to take during an asthma attack was compared separately against guidance on initial medical management for those aged two and older [5].

**Table 1:** Items that should be addressed by comprehensive asthma self-management education materials

Topic	Criteria
Basic facts about the nature of the condition	States that asthma is a lung disease characterized by inflammation and narrowing of the airways States that the four main symptoms of asthma are cough, wheeze, shortness of breath and chest tightness States that asthma cannot be cured (although childhood symptoms may remit) but can be effectively controlled States that the cause of asthma is not known

The nature of treatment: relievers and preventers	States that there are two classes of medication: relievers and preventers Explains possible side effects of medication (tachycardia/tremor in Β_2 _agonists; thrush/cataracts/dysphonia for inhaled steroids; possible additional effects for high dose steroids) States that early treatment can prevent symptoms from worsening

Allergen and trigger avoidance	States that recognizing and avoiding personal triggers is an important part of asthma control Provides guidance consistent with the primary and Secondary prevention components of the BTS/SIGN guidelines in relation to specific triggers

How to use treatment	States that preventer medication must be used regularly to be effective States the importance of good inhaler technique and appropriate use of a spacer device States the importance of ensuring inhalers are in date and are not empty

Self-monitoring and assessment skills	States that learning to recognize signs of change in asthma symptoms is an important personal skill States that all patients with asthma should have a peak flow meter Explains the purpose of a peak flow meter and how to use it States the importance of regular physician review

The role of a written, personalized action plan	States that patients with asthma should have an up to date written action plan. Explains the purpose of an action plan (to step up and step down treatment, and to seek appropriate help in response to changing symptoms and/or peak flow)

Recognizing and responding appropriately to acute exacerbations	Describes signs/symptoms of worsening asthma (increasing wheeze; cough; night time disturbance breathlessness limiting activity; reliever inhalers not working) States the importance of changing treatment and/or seeking help promptly Lay management of acute asthma

Personalizing the definition of good asthma control	States that it is reasonable for most people to achieve minimal symptoms and limitation of activities Asks patients to reflect on what they would consider as good asthma control Advocates discussion with personal health provider to set treatment goals in partnership

Topics were based on UK BTS/SIGN, US EPR-3 and GINA guidelines.

**Table 2:** Evidence-based statements extracted from international guidance used to assess compliance with evidence-based recommendations.

Statement	Evidence-base	Rationale for categorization
Secondary prevention-removal of pets from the home	Uncertain	"Complete avoidance of pet allergens is impossible [...] Although removal of such animals from the home is encouraged, even after permanent removal of the animal it can be many months before allergen levels decrease and **the clinical effectiveness of this and other interventions remains unproven**." [7] "**The reported effects of removal of pets from homes are paradoxical**, with either no benefit for asthma, or a potential for continued high exposure to induce a degree of tolerance." [5] [EPR-3 suggests that animal removal could be considered but rates the evidence Grade D which reflects panel consensus only] [6]

Secondary prevention-fungal allergen avoidance and control measures	Uncertain	"Air conditioners and dehumidifiers may be used to reduce humidity to levels less than 50% and to filter large fungal spore. **However, air conditioning and sealing of windows have also been associated with increases **in fungal and house dust mite allergens." [7] "Although fungal exposure has been strongly associated with hospitalisation and increased mortality in asthma, **no controlled trials have addressed the efficacy of reduction of fungal exposure **in relation to control of asthma." [5] "The Expert Panel recommends consideration of measures to control indoor mold [...] but **the relative contribution of fungi, house-dust mites or irritants [to asthma symptoms] is not clear**." [6]

Secondary prevention-cockroach avoidance and control measures	Uncertain	"[Measures for cockroach control] are **only partially effective in removing residual allergens**." [7] "Cockroach allergy is not a common problem in the UK and studies of attempts to avoid this allergen elsewhere have **produced conflicting results**." [5] [EPR-3 recommends cockroach control **if the patient is sensitive to cockroaches**]. [6]

Secondary prevention-cessation of active smoking	Beneficial	"Secondhand smoke **increases the frequency and severity of symptoms **in children with asthma." [7] "Direct or passive exposure to cigarette smoke **adversely affects **quality of life, lung function, need for rescue medications for acute episodes of asthma and long term control with inhaled steroids." [5] "[Smoke exposure] is associated with **increased symptoms, decreased lung function, and a greater use of health services **among those who have asthma." [6]

Secondary prevention-avoidance of passive smoking	Beneficial	"Asthma patients who smoke and are not treated with inhaled glucocorticosteroids, have a greater decline in lung function than asthmatic patients who do not smoke." [7] "Direct or passive exposure to cigarette smoke **adversely affects **quality of life, lung function, need for rescue medications for acute episodes of asthma and long term control with inhaled steroids."[5] "[Smoke exposure] is associated with **increased symptoms, decreased lung function, and a greater use of health services **among those who have asthma." [6]

Secondary prevention-avoidance of exposure to air pollution	Uncertain	"Avoidance of unfavourable environmental conditions is **usually unnecessary **for patients whose asthma is controlled." [7] "While it might seem likely that moving from a highly polluted environment might held, in the UK, **asthma is more prevalent in 12-14 year olds in non-metropolitan rather than metropolitan areas**." [5] "Clinicians [should] advise patients to avoid, to the extent possible, exertion or exercise outside when levels of air pollution are high." [6]

Secondary prevention-immunotherapy for a defined allergen	Beneficial	"Appropriate immunotherapy requires the **identification and use of a single well-defined clinically relevant allergen**." [7] "Immunotherapy can be considered in patients with asthma where a **clinically significant allergen cannot be avoided**." [5] "Immunotherapy [should] be considered for patients who have persistent asthma if evidence is clear of a **relationship between symptoms and exposure to an allergen**." [6]

Secondary prevention-weight reduction in obese patients	Beneficial	"Weight reduction in obese patients with asthma has been demonstrated to **improve lung function, symptoms, morbidity and health status**." [7] "One randomised parallel group study has shown **improved asthma control following weight reduction **in obese patients with asthma." [5] "Obesity has been associated with **asthma persistence and severity **in both children and adults. [...W]eight loss in adults resulted in improvement in pulmonary mechanics, improved FEV1, reductions in exacerbations and courses of oral corticosteroids and improved quality of life." [6]

Secondary prevention-seasonal influenza vaccination	Uncertain	"Patients with moderate to severe asthma should be advised to receive an influenza vaccination every year [...] **however routine influenza vaccination of children and adults with asthma does not appear to protect them from asthma exacerbations or improve asthma control**." [7] "Immunisations should be administered **independent of any considerations related to asthma**." [5] "[We recommend] that clinicians consider inactivated influenza vaccination for patients who have asthma [...] however the vaccine should **not be given with the expectation that it will reduce either the frequency or severity of asthma **exacerbations during the influenza season." [6]

Statements were extracted from the UK BTS/SIGN, US EPR-3 and international GINA guidelines.

*'*Uncertain' was used when either all three guidelines agreed that the evidence base was uncertain or where the advice given by the guidelines differed. We retained only topics discussed by all three sets of guidelines.

We used the US National Center for Complementary and Alternative Medicine definition of Complementary and Alternative Medicine (CAM) [10] to define a group of apps that were not assessed for either comprehensiveness or consistency with evidence since these concepts are not consistently recognized in CAM practice. However, we retained these apps within the overall descriptive summary of app types and, specifically, for assessment of emergency management advice because of the particular risks associated with acute asthma.

In addition to those with information content, we anticipated that we would find apps offering diary features and planned to assess compliance with the recommendations of a recent systematic review concerning the components of self-management plans [11]. We also assessed apps using a set of content-independent quality criteria that we derived from an existing set of criteria for website-based health information developed by the Health on the Net foundation [12] (Table 3). These define a set of eight best-practice principles relating to attribution, traceability and transparency of information. For apps that lacked attribution, we used general purpose internet search engines (Google, Google Incorporated, Mountain View, CA, USA and Bing, Microsoft Corporation, Redmond, WA, USA) to attempt to locate original authors for written content.

**Table 3:** Health information best-practice principles adapted for smartphone apps

1	Information must be authoritative: all medical information presented by [and/or calculations performed by an app] must be attributed to an author and his/her training in the field must be mentioned.
2	Purpose [of the app]: A statement clearly declaring that the [app] is not meant to replace the advice of a health professional has to be provided. A brief description of the [app]'s mission, purpose and intended audience is necessary. Another brief description of the organisation behind the [app], its mission and its purpose is also necessary.

3	Confidentiality: The [app publisher] must describe its privacy policy regarding how you treat confidential, private or semi-private information such as email addresses and the content of emails received from or sent to [its users]

4	Information must be documented, referenced and dated: All medical content [including calculations and formulae] has to have a specific date of creation and a last modification date.

5	Justification of claims: All information about the benefits or performance of any treatment (medical and/or surgical), commercial product or service are considered as claims. All claims have to be backed up with scientific evidence (medical journals, reports or others).

6	[App] contact details: The [app] must be operational and the information must be accessible and clearly presented. There must be a way to contact the [app publisher], such as a working email address or contact form, for visitors who would like to have more details or support.

7	Funding: [The app publisher] must include a statement declaring its sources of funding.

8	Editorial and advertising policy: Conflicts of interest and external influences which could affect the objectivity of the editorial content must be clearly stated in the disclaimer. All [apps] displaying paying banners have to have an advertising policy. This policy must explain how the [publisher] distinguishes between editorial and advertising content and which advertisements are accepted. Any conflict of interest has to be explained.

Adapted from the Health on The Net Foundation principles for health information on the internet.

Each reviewer recorded their responses in a structured form. These were compared and any discrepancies were resolved by discussion. Throughout the assessment process, we kept a record of any problems using the software that were encountered using a general schema to classify errors (Table 4).

**Table 4:** Classes of software issue considered during assessment

Issue type	Description	Example(s)
Data entry validation	Data can be entered that are out-of-range or inappropriate. New data can overwrite existing data without warning.	Negative values of peak flow can be entered and are stored. New entries can overwrite existing data without warning.

Functionality	A function of the app (for example, saving data, performing a calculation) does not operate as expected.	App miscalculates the score of Asthma Control Test for adults; app displays an 'unfortunately you did not beat your highest score' message even if score is 100%.

Presentation and user interface (UI)	Content having spelling and layout mistakes. User interface controls (for example, textboxes, labels, buttons) are mislabelled, inoperative or inaccessible. Navigation between different parts of the app does not occur as expected or can lead to the user getting stuck on a particular screen.	Some controls hidden when opened on a lower resolution screen; text box for recording peak flow labelled as 'Peek Flow'; some user controls not labelled in English.

Crash	The app stopped responding in a timely way to user input or was closed unexpectedly by the smartphone or tablet operating system.	App crashed when a backup of data entered by the user was attempted.

Other	Any other software issue, for example an online or other data service (for example, a website that the app uses for data) is unavailable or does not work as expected.	Some linked content that is displayed within the app on allergens is broken; GPS function does not work.

The table summarizes the classes of error used for assessment and provides illustrative examples based on those observed during the appraisal process.

#### Post-hoc assessments

An additional analysis was performed to assess the properties of calculator and questionnaire-based apps that were not anticipated during study planning. In each case, we looked for evidence of external validation [13]. We also tested the calculators and any questionnaire scoring to confirm that these were numerically correct.

### Statistics

Descriptive statistics were used to summarize the results of the content assessment.

## Results

**Figure 2:**
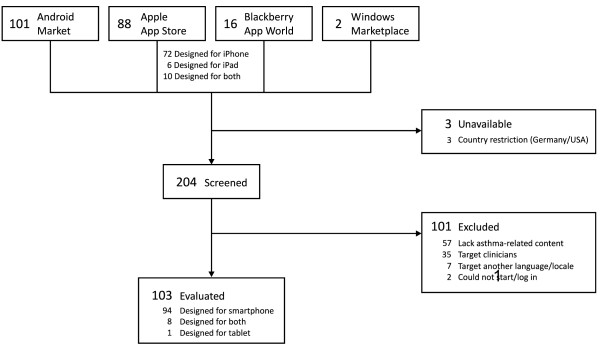
**Flowchart of app selection process**.

Searches performed in August 2011 identified 207 apps from the app stores for Android, Apple, Blackberry and Windows Phone, of which 204 were available for screening (Figure 2). We excluded 101 that either contained no health or asthma-related content (n = 57), targeted clinicians (n = 35), were not in English (n = 7), or could not be started (n = 2). Excluded apps are summarized in Additional file 2. Subsequent discussion is restricted to the 103 apps that met inclusion criteria. Most (n = 94) were designed for smartphones. Although all iPhone apps can run on the iPad tablet, we found eight that included specific customizations to take advantage of the large screen size and one that was a tablet-only app.

### Summary of characteristics of included apps

Fifty-six apps were sources of written (n = 43) and multimedia (n = 13) information about asthma and its management [14-69]. The remaining 47 were tools supporting aspects of asthma self-management and included diaries (n = 29), assessment instruments (n = 17) and location-based alerts (n = 6) [70-115]. Although we neither expected nor required that assignment into these two main categories be mutually exclusive, we found no English-language apps that combined both information and management tools. (A typology is provided in Additional file 3.) Sixty-one percent (n = 34) of information apps and 96% (n = 45) of management tools focussed on conventional medical management of asthma. Nineteen apps considered exclusively CAM, while five integrated content addressing both approaches. Seven apps targeted either younger children [38,89] or their parents [16,27,106-108]. None targeted adolescents or elderly patients.

After including any in-app purchases required to access asthma-specific content, the majority of apps (n = 76/103) were not free with a median cost of £1.49 (mean £1.85, range £0.61 to £8.99). Apps offering management tools were more likely to be free (n = 19/47) compared to those presenting health information (n = 8/56).

### Apps presenting health information

Apps presenting health information are summarized in Additional file 4.

#### Comprehensiveness of asthma information

Excluding apps exclusively addressing CAM, 38 apps were evaluated for comprehensiveness of asthma information [14-22,24-41,43,48,49,54-56,58,59,63,64,67]. The basic nature of asthma, including the role of inflammation, symptoms and prognosis was the most commonly addressed (at least partially), by two-thirds of apps (n = 26, breakdown in Additional file 5). Allergen and trigger avoidance were discussed by 18 but only covered in depth by 2 [25,63]. Less than two-fifths addressed recognition of exacerbations (n = 14), self-monitoring (n = 10) and inhaler techniques (n = 10). Customized aspects of asthma management, including the role of an action plan and the prioritization of treatment goals according to patient wishes, were addressed least frequently by seven and three apps, respectively. Three apps wholly addressed six of the eight domains and provided partial coverage of the remaining two [19,59,63].

#### Lay management of acute asthma

Of the 14 medical apps containing conventional information about recognition and management of acute asthma [20,25,28,29,33-37,49,58,59,63,64], 7 provided specific guidance on lay management of an asthma attack. Although none addressed all aspects of the step-wise approach recommended by guidelines, six gave advice that was broadly consistent [33-36,49,63] but lacked specific instructions on the dose and frequency of reliever inhaler use (addressed by n = 2/6) or the appropriate medical service to contact (addressed by n = 2/6). One app provided guidance that substantially differed from recommendations [29].

Eight apps suggested CAM procedures for acute asthma management [35,36,48,51,52,60,62,65]. None recommended using a beta-agonist reliever inhaler or seeking conventional medical help should an alternative emergency procedure prove ineffective, although two contained details of conventional emergency management in separate sections [35,36].

#### Compliance of information with evidence-based recommendations

We identified 72 instances where apps addressed items from our pre-defined set of evidence-based recommendations. Of these app-statements, 40 were asserted in line with current guidance. In all other cases (n = 32), apps appeared to unequivocally recommend a particular course of action where there is current uncertainty. Statements concerning active and passive smoke avoidance (exacerbates symptoms, n = 12 and 13), weight reduction in obesity (beneficial for asthma symptoms, n = 7) and the potential utility of immunotherapy (can be considered where a specific allergen is identified, n = 2) were correctly asserted by all apps that mentioned them. Recommendations about behavioural strategies for the avoidance of air pollution (n = 9), fungal allergens (n = 9), removal (rather than control of) pets (n = 5) and cockroach control (n = 7) were all delivered more variably (breakdown in Additional file 6). An unequivocal recommendation for flu vaccination was made by five of six apps.

A small number of apps actively cautioned against allopathic medical management. Four apps [18,52,60,66] recommended avoiding conventional medical management because of the risks of side effects, addiction and worsening of the condition.

### Apps providing tools for the management of asthma

#### Diaries

Twenty-nine apps offered functions for patients to track their asthma (Additional file 7[70-98]). Diaries differed in terms of the information that they captured and the options given to patients for manipulating the recorded data. While a small number of diaries captured either asthma symptoms (n = 2 [87,88]) or peak flow (n = 2 [93,94]) alone, the majority (n = 23) allowed both symptoms and peak flow values to be recorded as well as recent medication use (n = 24). Most apps relied on manual entry of data; however, one [92] was able to source values from a Bluetooth-enabled peak flow meter and another (available on both iPhone and Windows Phone) from a wireless inhaler [96,97] (untested in this review). Fifty-nine percent of (n = 17 of 29) diary apps lacked data validation to prevent out-of-range values to be entered [71,73-77,79-82,86,90,93-96,98]. Five diaries allowed customized self-management plans [76,77,84,89,92] that included emergency care instructions and prescribing details for different classes of medication. Four [76,77,84,92] used a three-step action plan with traffic light colouring consistent with guideline recommendations [11]. However, none were able to vary the number of steps in the action plan, nor the thresholds at which the action plan steps were triggered (50 and 80%). All four used peak flow values entered in the diary to trigger a display of steps to be taken by the patient based on their action plan. Although recommended by guidelines, none included an equivalent function based on recorded symptoms.

Five apps [82,90,95-97] provided a function to track the doses remaining in their pressured Metered-Dose Inhaler (pMDI). Each app used a similar approach, providing a visual warning when the device was running low.

#### Assessment instruments

The sources and scoring mechanisms of asthma status questionnaires embedded in seven [82,89,99,100,106-108] were reviewed (Additional file 8). Only one app cited the source [82], assigning a numeric score based on Global Initiative for Asthma criteria for asthma control [7]. However, while these criteria exist [7], we could find no validated approach that recommends assigning a numeric score to each criterion and presenting the result as an aggregate sum. One [99] used, without attribution, the adult and paediatric versions of a standard instrument, the Asthma Control Test [116,117]. Scoring errors were found in the adult version, which meant that no matter how minimal an individual's current symptoms, the app would always recommend seeking medical help. We could not find validation information for any of the other tools.

Three iPhone apps [101-103] used the device microphone to analyse breath sounds and provided diagnostic commentary, for example, the identification of wheeze. We were unable to locate validation information for these diagnostic tools.

Seven apps incorporated predicted peak flow calculators as either a dedicated calculator (n = 3 [104,105,118]) or within a diary to generate reference values for charting (n = 4 [80,81,83,94]). Only one of the calculators [118] provided attribution. We were able to identify the calculation algorithm for one other [105]. Both had bugs which resulted in incorrect output being generated under certain circumstances. One [105] would silently forget the gender of the patient and subsequently provide male predicted values if the device was physically rotated to change from a portrait to landscape screen display. The other had a systematic error where female predicted values were returned for individuals five inches shorter in height than those entered [118]. Despite writing to the publishers, we could not identify the underlying algorithm for the third calculator or any but one of the diary apps [83], the performance of which could not be verified because of problems entering data. Only one acknowledged the use of different peak flow measurement scales by allowing the user to pick which type of meter they used [94].

#### Other tools

Six apps provided location-based pollen or pollution alerts for users in the United States and Ireland [70,109-113] (Additional file 9). One product - available as apps on both Blackberry [115] and Apple [114] devices - did not fit into the categories of tools described above, offering paid-for audio recordings of Indian chants intended for use by those with a range of conditions including asthma.

### Compliance with health information best-practice principles

The purpose of the app was clearly stated or interpretable in 86% of health information apps (n = 48) and 96% of management tools (n = 45). Content authorship was stated in 18 of 56 (32%) health information apps. Six apps [19,29,35,36,48,66] were eBook versions of texts originally available in hard copy. Where information was not attributed, we searched online in an attempt to locate any original source. A quarter of information apps (n = 14, of which 10 were paid for) used content available freely online without attribution, for example, from Wikipedia [20,25]. In a further five cases [18,31,33,34,57], we found matched content online but it was unclear whether reproduction was authorized. The date of content creation was identified for only one app [27] and none provided a content expiry date. Only one provided details of its editorial policy through a linked website [27].

An explicit confidentiality policy - found either in the app or on an associated website - was identified for only 5 of 29 apps (17%) in which personal data could be recorded [70,72,82-84]. Four apps offered a password protection mechanism to assist in securing data [78,83,84,96]. We were able to identify the funding source for the app in 23 cases: 2 were sponsored by local US government [109,110]; one medication tracker [95] (and 2 German-language apps excluded from the analysis [119,120]) were sponsored by pharmaceutical companies; 2 by a company developing an electronic inhaler [96,97] and the rest by commercial companies Twenty-two apps incorporated advertisements but none detailed an advertising policy. Most (n = 17) were for products unrelated to health and the remainder promoted content offered by the same publisher. Third party endorsements were present for two apps; from the US National Institutes of Health [59] and the UK Department of Health [27], under the Information Quality Mark scheme. Fifty-five percent of all apps offered a means to contact the authors using either email (n = 41), an online form (n = 14) or an in-app form (n = 2).

## Discussion

We systematically assessed all available asthma apps to ascertain whether they would be appropriate for prescription by health professionals by meeting existing quality standards for asthma self-management information and tools. Although our search identified 103 English language asthma apps, none combined comprehensive, evidence-based information with reliable supportive tools.

While the majority of information apps presented incomplete content that does not address the breadth of topics recommended for people living with asthma, a few did. Asthma Consultant for Blackberry [59], Truth About Asthma for Android [63] and Asthma for iPhone [19] addressed the widest range of topics and provide guidance consistent with US guidelines. Focussing on childhood asthma, Child Health for iPhone [27] is noteworthy because it most completely complied with standards for the presentation of health information. No apps provided comprehensive advice about lay management of acute asthma and a small number of CAM apps made recommendations that are likely to be ineffectual or may be harmful.

Inconsistencies were also seen in apps that offered tools. Peak flow and symptom diaries, although generally reliable, often lack basic features, such as data entry validation, and, consequently, no particular app stood out in this group. Peak flow calculators and questionnaire-based apps designed for use by people with asthma (as distinct from those targeting clinicians which were not assessed in this review) appear to be unreliable and should not be used: we were unable to identify the underlying calculation for most; numeric errors were present in those that we could verify and only one attempted to compensate for differences in readings from different peak flow meter types.

These findings have a number of consequences for clinicians, who may be considering using apps as part of routine asthma care. First, no current app can meet the needs of every patient. Instead, clinicians will need to draw from the diverse range of options. This requires that they themselves become familiar with a large number of apps, or that appropriate guidance is available to them. Second, because information apps have different levels of information coverage, they must identify, or have identified for them, the gaps in those apps that are in use and tailor their education to address these. Third, clinicians must consider how best to support people who choose their own app, particularly where the advice it contains differs from current practice. The potential complexity of these tasks suggests that there may be something of a missed opportunity to combine comprehensive information and tools in a single app.

Health professionals, particularly GPs with very limited consultation time, face broader issues in prescribing apps that function as self-management tools. Patients may need help addressing technical issues, such as installing an app or fixing it if it crashes. Apps that enable the creation of action plans or data collected into diaries require new ways of working to populate those records, review data and respond in a timely fashion. If data are being transferred from patient devices, clinicians must think about how those data will be integrated into patient records and their responsibilities for data security.

The quality gaps we identified in asthma apps, and similar findings for iPhone apps for smoking cessation [121] and weight loss [122] available in 2009, should prompt caution in health professionals and policy makers thinking about the imminent introduction of apps into long-term condition care. While we recognize that apps are, and will remain, a rapidly developing field, and that higher quality apps may (always) be available in the near future, the classes of issues we have identified will persist. They serve to highlight some of the actions necessary to translate apps from opportunity to implementation. Specifically, mechanisms of quality control and greater conceptualization and validation of the role of apps in clinical care need to be addressed.

There are many existing mechanisms for quality assurance in medicine that could be applied to apps. As we have shown, guidelines and standards for condition-specific information and information-use more generally exist and can provide criteria against which to assess app content. Similarly, for each condition, validated tools exist that can be appropriated (with appropriate attribution), allowing assurance to focus on correct implementation rather than the content of the tool. Processes from medical device regulation could provide guidance on ways to tackle quality assurance of technological aspects, such as the reliability and data validation problems highlighted in this review.

Guideline-derived quality criteria, such as ours, have a rational basis from a clinical point of view but have limitations. First, the modes of delivery envisaged when guidelines were written may be a poor match with apps. For example, paper-based asthma action plans lack the interactive treatment recommendations offered by most of the app-based plans in our review. Second, some quality issues may not result in actual harm, either because they are readily detected or because their effects are minimal in practice. Quality may also be judged differently by patient users of the app, who must find that a specific app meets their own goals and stimulates sustained use. Early evidence is equivocal about whether mobile apps for asthma self-management are better than existing paper-based methods: of the two medium-sized randomized trials identified as part of an on-going systematic review [123], one, a Taiwanese trial of an asthma diary app demonstrated reductions in exacerbations and unscheduled care use [124] while the second, a UK-based trial of a similar app, did not [125]. Future work should seek to understand the range of dimensions that play a part in making apps effective. Promoting reliable, evidence-based apps is only the starting point to exploring the role of apps as part of long-term condition care.

Policy makers will need to consider the best ways to combine existing appraisal processes or build new ones to promote app quality assurance in all of its angles. Possibilities include making app assessment part of the remit of guideline groups, requesting systematic reviews of content similar to this paper, self- or third-party accreditation, full regulation, usage studies and recommender systems. Any of these solutions will need to account for the dual issues of content assessment and technology reliability and safety which have traditionally been done by different groups. They will also need to attend to the tension between the desire to limit risk to patients and the barrier that assurance creates in terms of cost and effort in what is, and will remain, a rapidly evolving field. This tension is not new. Commentary on previous technology innovations, for example, the emergence of the internet as a source of health information, has highlighted the risk of mismatch between standards proposed for new media and those seen in existing media [126,127]. Any assurance strategy should be demonstrably effective in helping clinicians - and people with asthma - identify high quality apps appropriate to their needs.

## Conclusions

Little evidence exists around the content quality of apps for long-term conditions, although limited adherence to evidence-based guidelines has been found in apps for behavioural interventions, like smoking and weight loss. This systematic assessment of apps for asthma provides insight into the types of quality issues that can affect apps for long-term conditions as well as a replicable and updatable method for assessment. Issues include limited and inaccurate information, faulty tools and content-independent problems of information presentation and attribution. The findings highlight a need for caution among clinicians thinking about using apps as part of clinical care and for policy-makers and developers to consider appropriate ways of assuring content quality in health apps. There is much to be done: clinicians cannot recommend tools that are inaccurate, unsafe or lack an evidence base.

## Abbreviations

BTS/SIGN: British Thoracic Society/Scottish Intercollegiate Guideline Network; CAM: Complementary and Alternative Medicine; EPR-3: Expert Panel Report 3; GINA: Global Initiative for Asthma

## Competing interests

KH's doctoral studies, of which this work formed part, are funded by the NIHR CLAHRC in North West London. CP is funded by the NIHR CLAHRC for Peterborough and Cambridge. No financial relationship exists with any organizations that might have an interest in the submitted work within the previous three years. There are no other relationships or activities that could appear to have influenced the submitted work.

## Authors' contributions

KH and JC conceived the study. KH designed the data extraction process and carried out the searches. KH and MC screened and appraised the included apps. KH took lead responsibility for drafting the article and CM, MC and JC had a significant role in its critical revision. JC had full access to the data in the study and acts as guarantor for the study. All authors read and approved the final manuscript.

## Pre-publication history

The pre-publication history for this paper can be accessed here:

http://www.biomedcentral.com/1741-7015/10/144/prepub

## Supplementary Material

Additional file 1 **Data extraction template**. The template was used during data extraction to standardize the process. Descriptions are provided for items that may be unclear. Section 8 contains a comprehensive list of evidence-based statements derived from UK BTS/SIGN guidelines which is a superset of those reported in the study and is intended for a separate analysis of the suitability of apps for use in a UK-specific context. Click here for file

Additional file 2 **Characteristics of excluded apps**. The table summarizes the apps excluded during screening and the reasons for exclusion. Click here for file

Additional file 3 **App types identified by the systematic assessment**. The table shows a numeric breakdown of the types of content and features exposed by apps included in the assessment. Click here for file

Additional file 4 **Characteristics and quality appraisal of apps presenting information about asthma**. Methods used for quality appraisal are described in the text. Click here for file

Additional file 5 **Comprehensiveness of educational topic coverage by health information apps**. The table summarizes the number of apps addressing each of eight topics defined for asthma self-management education. Each app was assessed as either addressing the domain wholly, partially or not at all, using defined sub-criteria, which are described separately. Click here for file

Additional file 6 **Consistency of recommendations made by asthma apps with evidence-base**. The table shows the number of references to each statement made by apps included in the assessment and the direction of any recommendation associated with that statement. For example, an app claiming that removing pets from the home is beneficial for asthma symptom control would count under the 'Beneficial' column. The expected advice (in the example given: that there is no clear evidence that removal of pets from the home improves asthma symptoms) is shown for each statement in the shaded box. Click here for file

Additional file 7 **Characteristics and quality appraisal of diary apps**. Methods used for quality appraisal are described in the text. Click here for file

Additional file 8 **Characteristics and quality appraisal of apps providing assessment tools**. Methods used for quality appraisal are described in the text. Click here for file

Additional file 9 **Characteristics of other apps**. The table summarizes apps not fitting into other categories. Click here for file
