# Supplementary material for: Apps for asthma self-management: a systematic assessment of content and tools
Source: BMC Med. 2012 Nov 22;10:144. doi: 10.1186/1741-7015-10-144 (PMC3523082; doi:10.1186/1741-7015-10-144)
Supplement: Additional file 1 — Data extraction template. The template was used during data extraction to standardize the process. Descriptions are provided for items that may be unclear. Section 8 contains a comprehensive list of evidence-based statements derived from UK BTS/SIGN guidelines which is a superset of those reported in the study and is intended for a separate analysis of the suitability of apps for use in a UK-specific context. [file 1741-7015-10-144-S1.DOC]

# Additional File 1

Items in structured data extraction form

The template was used during data extraction to standardise the process. Descriptions are provided for items that may be unclear. Section 8 contains a comprehensive list of evidence-based statements derived from UK BTS/SIGN guidelines^1^ which is a superset of those reported in the study and is intended for a separate analysis of the suitability of apps for use in a UK-specific context.

| **ID** | **Item** | **Format** | **Description** |
| --- | --- | --- | --- |
| 0.1a | App ID | Number | Internal identifier assigned to track apps during review process. |
| 0.1b-d | Linked IDs | Number | Identifier of related app, e.g. free/paid versions, same app on a different platform, etc. |
| 0.2 | Date of review | Date | Date on which review was performed. |
| 0.3 | Reviewer | Text | Name of reviewer. |
| 0.4 | Downloaded | Yes/No | Whether app was successfully downloaded. |
| 0.5a | Platform | Pick | Platform on which app runs. |
| 0.5b | Is tablet? | Yes/No |  |
| 0.6a | Include or Exclude? | Include/Exclude | Assessment of whether app meets inclusion criteria. |
| 0.8 | Typology | Text | Categories of functionality exposed by app, evolved by reviewers during assessment process; includes record of why apps were excluded. |
| *Basic details* |  |  |  |
| 1.1 | App name | Text |  |
| 1.2a | Version | Number |  |
| 1.2b | First release? | Yes/No |  |
| 1.3a | Release date | Date |  |
| 1.3b | Release quarter | Quarter |  |
| 1.4a | Cost | £ or 'Free' |  |
| 1.4b | Addon Cost | £ | Cost of additional features that could be ‘unlocked’ within the app for a fee. |
| 1.4c | Addon Feature | Text | Description of additional features. |
| 1.4d | TCO | £ | Total cost of obtaining a fully functional version of the app |
| 1.5 | Publisher | Text | Name of software publisher. |
| 1.6 | Source country | Text | Source country identified by tracing details of software publisher. |
| 1.7 | Website | Link |  |
| 1.8a | Number of downloads | Number or Number Range | Download data sourced from app marketplaces; not available for all platforms. |
| 1.8b | Download count census date | Date | Date on which download count was sourced. |
| 1.8c | Average user rating | Number | User rating data sourced from app marketplaces; not available for all platforms. |
| 1.8d | Number of user ratings | Number |  |
| 1.9 | Language | Text | Language(s) available for user interface and content within app. |
| *Functionality* |  |  |  |
| 2.1 | Main function | Text | Main functions exposed by the app as claimed by the software publisher in their app promotional materials |
| 2.2 | Brief description | Text | Summary of app for internal use as aide-memoire. |
| 2.3a | Stated or implied target | Clinician/Patient/Parent/Other | Target of app as claimed by software publisher. |
| 2.3b | Interpreted target | Clinician/Patient/Parent/Other | Target of app as interpreted by reviewer when no claim of target made by publisher. |
| 2.4a | Is the app intervention standalone? | Yes/No | Yes/No depending on whether the app is designed to fit into a system of care or be used with other tools, e.g. a website. |
| 2.4b | If 2.4a NO, provide details of intervention | Text |  |
| 2.5 | Is there a user setup wizard? | Yes/No | Yes/No depending on whether the app provides a structured process to assist users when first running the app. |
| 2.6 | Does the app support multiple user profiles? | Yes/No | Yes/No depending on whether multiple users can store their details in the app. |
| 2.7a | What data services does the app demand | Location/Push/Cell/Email/Data |  |
| 2.7b | Requires network connectivity to work? | Yes/No | Yes/No depending on whether major functions of the app will not work if there is no connection to the internet. |
| 2.7c | Uses Google Health? | Yes/No |  |
| *Diary features* |  |  |  |
| 3.1 | Does the app have diary features? | Yes/No |  |
|  | *If 3.1 NO, skip to the section 4* |  |  |
| 3.2a | Does the app have a diary to record peak flow readings (PEFR)? | Yes/No |  |
| 3.2b | If 3.2a YES, provide details | Text |  |
| 3.2c | If 3.2a YES, does the app use a predicted or % of personal best to classify readings? | Predicted/Personal Best/Both/No |  |
| 3.2d | If 3.2c PREDICTED, what formula is used to calculate predicted PEFR? | Text or 'Unknown' |  |
| 3.2e | If 3.2a YES, is there a PEFR summary visualisation? | Yes/No | Yes/No if there is a graph, table or chart summarising longitudinal data, trends or statistics relating to PEFR. |
| 3.2f | If 3.2e YES, provide details | Text |  |
| 3.3a | Does the app have a diary to record symptoms? | Yes/No |  |
| 3.3b | If 3.3a YES, provide details | Text |  |
| 3.4a | Does the app have a diary to record medication? | Yes/No |  |
| 3.5a | If 3.2a or 3.3a YES, does the app provide any alert based on changing PEFR/symptoms? | Yes/No | Yes/No depending on whether the app displays a popup, warning or other notification that is tailored by data entered by the user. |
| 3.5b | If 3.2a or 3.3a YES, does the app provide any advice based on changing PEFR/symptoms? | Yes/No | Yes/No if the warning message contains advice, e.g. to change treatment, seek help, etc. rather than simply notify of the change. |
| 3.5c | IF 3.5b YES, what advice is given? | Text |  |
| 3.6a | Does the app allow data to be shared with a clinician? | Yes/No |  |
| 3.6b | If 3.6a YES, provide details | Text |  |
| *Alerts* |  |  |  |
| 4.1a | Does the app have a pollen alert? | Yes/No |  |
| 4.1b | Does the have a pollution alert? | Yes/No |  |
| 4.2a | Does the app provide any other reminders? | Yes/No |  |
| 4.2b | If 4.2a YES, provide details | Text |  |
| *Information* |  |  |  |
| 5.1 | Does the app provide any information or guidance about asthma? | Yes/No |  |
| *Action plan* |  |  |  |
| 6.1a | Does the app recommend the use of a personal asthma action plan? | Yes/No |  |
| 6.1b | Does the app have a feature to store personal asthma triggers? | Yes/No |  |
| 6.1c | Does the app have a feature to store a personal asthma action plan? | Yes/No |  |
|  | *If 6.1c NO, skip to section 7* |  |  |
| 6.2a | Does the plan match the 4-stage template used by in the UK? | Yes/No |  |
| 6.2b | If 6.2a NO, how does it differ? | Text |  |
| 6.3a | Does the app use the plan to make treatment recommendations? | Yes/No |  |
| 6.3b | If 6.3a YES, provide details | Text |  |
| *Emergencies* |  |  |  |
| 7.1 | Does the app provide advice on what to do during an asthma attack? | Yes/No |  |
|  | *If 7.1 NO, skip to section 8* |  |  |
| 7.2a | If 7.1 YES, does the advice match guidelines (e.g. Asthma UK attack card)? | Yes/No |  |
| 7.2b | If 7.2a NO, how does it differ? | Text |  |
| *Complying with current guidance* | *Does the app recommend any of the following…* |  |  |
| 8.1a | Primary prophylaxis - dust mite aeroallergen avoidance | Pick (Beneficial, Not Beneficial, Unclear) |  |
| 8.1b | Primary prophylaxis - maternal food allergen avoidance during pregnancy and lactation | Pick |  |
| 8.1c | Primary prophylaxis - breast feeding | Pick |  |
| 8.1d | Primary prophylaxis - use of modified infant milk formulae | Pick |  |
| 8.1e | Primary prophylaxis - timing of weaning | Pick |  |
| 8.1f | Primary prophylaxis - nutritional supplementation with fish oils during pregnancy | Pick |  |
| 8.1g | Primary prophylaxis - other nutritional supplements during pregnancy | Pick |  |
| 8.1h | Primary prophylaxis - nutritional supplementation with dietary probiotics during pregnancy | Pick |  |
| 8.1i | Primary prophylaxis - avoidance of tobacco smoke and air pollutants during pregnancy | Pick |  |
| 8.1j | Primary prophylaxis - avoidance of tobacco smoke and air pollutants during early childhood | Pick |  |
| 8.1k | Primary prophylaxis - immunotherapy in childhood | Pick |  |
| 8.1l | Primary prophylaxis - normal course of childhood immunisation | Pick |  |
| 8.2a | Secondary prophylaxis - dust mite aeroallergen avoidance | Pick |  |
| 8.2b | Secondary prophylaxis - removal of pets | Pick |  |
| 8.2c | Secondary prophylaxis - fungal allergen avoidance | Pick |  |
| 8.2d | Secondary prophylaxis - cockroach avoidance | Pick |  |
| 8.2e | Secondary prophylaxis - cessation of active smoking | Pick |  |
| 8.2f | Secondary prophylaxis - avoidance of passive smoking | Pick |  |
| 8.2g | Secondary prophylaxis - avoidance of air pollution | Pick |  |
| 8.2h | Secondary prophylaxis - subcutaneous immunotherapy | Pick |  |
| 8.2i | Secondary prophylaxis - sublingual immunotherapy | Pick |  |
| 8.2j | Secondary prophylaxis - altering electrolyte intake (sodium, magnesium) | Pick |  |
| 8.2k | Secondary prophylaxis - nutritional supplements with fish oils/lipid | Pick |  |
| 8.2l | Secondary prophylaxis - nutritional supplements with antioxidants | Pick |  |
| 8.2m | Secondary prophylaxis - nutritional supplements with dietary probiotics | Pick |  |
| 8.2n | Secondary prophylaxis - weight reduction in obese patients | Pick |  |
| 8.2o | Secondary prophylaxis - normal course of immunisations | Pick |  |
| 8.2p | Secondary prophylaxis - flu vaccinations | Pick |  |
| 8.3a | Complementary therapy - acupuncture | Pick |  |
| 8.3b | Complementary therapy - air ionisers | Pick |  |
| 8.3c | Complementary therapy - yoga breathing exercises | Pick |  |
| 8.3d | Complementary therapy - Buteyko breathing exercises | Pick |  |
| 8.3e | Complementary therapy - herbal and traditional Chinese medicine | Pick |  |
| 8.3f | Complementary therapy - homeopathy | Pick |  |
| 8.3g | Complementary therapy - hypnosis | Pick |  |
| 8.3h | Complementary therapy - manual therapy, massage and spinal manipulation | Pick |  |
| 8.3i | Complementary therapy - physical exercise training | Pick |  |
| 8.3j | Complementary therapy - family therapy | Pick |  |
| 8.4a | Avoiding food additives | Yes/No |  |
| 8.4b | Adopting healthy exercise (with appropriate precautions for exercise-induced asthma) | Yes/No |  |
| 8.4c | The use of self-management as a way of improving symptoms? | Yes/No |  |
| 8.4d | Smoking composite | Yes/No |  |
| 8.4f | The regular use of preventative medicine? | Yes/No |  |
| 8.4g | The use of reliever medication when required? | Yes/No |  |
| 8.4h | The importance of good inhaler technique? | Yes/No |  |
| 8.4i | Avoiding standard approaches to managing asthma (i.e. not taking preventative medicine or relievers) | Yes/No |  |
| 8.4j | The use of drugs other than those recognised as part of asthma management in the BTS/SIGN guidelines (beta 2 agonists, inhaled steroids, etc) | Yes/No |  |
| 8.4k | The use of any other complementary medical technique not listed here (provide details) | Yes/No |  |
| 8.5 | If the answer to any of these questions is YES, provide details | Text |  |
| *Security and resilience* |  |  |  |
| 9.1 | Does the app offer password protection? | Yes/No |  |
| 9.2a | Does the app offer a backup mechanism | Yes/No |  |
| 9.2b | If 9.2a YES, provide details | Text |  |
| 9.3a | Were any software errors encountered during test use? | Yes/No |  |
| 9.3b | If 9.3a YES, provide details | Text |  |
| *Content and Attribution* |  |  |  |
| 10.1a | Does the app include adverts? | Yes/No |  |
| 10.1b | What kind of adverts does the app include? | Yes/No |  |
| 10.1c | Does the app offer paid-for enhancements? | Yes/No |  |
| 10.2a | Is there a disclaimer? | Yes/No |  |
| 10.2b | If 10.2a YES, quote appropriate text from disclaimer | Text |  |
| 10.3a | Are information sources attributed? | Yes/No |  |
| 10.3b | Provide details of attribution (or failure of attribution) | Text |  |
| 10.3c | Is information content dated? | Date |  |
| 10.3d | Is a content expiration date set? | Date |  |
| 10.4a | Is the app accredited or endorsed by any third party? | Yes/No |  |
| 10.4b | If 10.4a YES, provide details | Text |  |
| 10.4a | Does the app offer a local help system? | Yes/No |  |
| 10.4b | Does the app offer online help system? | Yes/No |  |
| 10.4c | Is there a support contact? | Yes/No |  |
| 10.4d | If 10.4c YES, provide details | Text |  |
| 10.5a | Were any textual/grammatical errors found? | Yes/No |  |
| 10.5b | If 10.5a YES, provide relevant example(s) | Text |  |
| *Other* |  |  |  |
| 11.1 | Notes | Text |  |
| 11.2 | Any other problems/issues identified | Text |  |
| *Education Domains* |  |  |  |
| 12.1 | Nature of disease | Yes/No/Partially | Judgement by reviewer based on criteria in Supplementary Table 2 |
| 12.2 | Nature of treatment | Yes/No/Partially |  |
| 12.3 | Appropriate allergen or trigger avoidance | Yes/No/Partially |  |
| 12.4 | How to use treatment | Yes/No/Partially |  |
| 12.5 | Development of self monitoring/assessment skills | Yes/No/Partially |  |
| 12.6 | Negotiation of personalised action plan in light of goals | Yes/No/Partially |  |
| 12.7 | Recognition and management of acute exacerbations | Yes/No/Partially |  |
| 12.8 | Areas where patient wants treatment to have effect: "If we could make one thing better for your asthma what would it be?" | Yes/No/Partially |  |
| *HON Domains* |  |  |  |
| 13.1 | Is the app purpose clear from the product advert? | Yes/No |  |
| 13.2 | If the app involves data collection, is there a confidentiality policy? | Yes/No |  |
| 13.3 | Does the app publisher disclose its funding model? | Yes/No |  |
| 13.4 | If the app has ads, does the app publisher have an advertising policy? | Yes/No |  |

^1^British Thoracic Society and Scottish Intercollegiate Guidelines Network (2011). British guideline on the management of asthma - a national clinical guideline. May 2008, Revised May 2011. Edinburgh, Scotland, SIGN.
