# Supplementary material for: Apps for asthma self-management: a systematic assessment of content and tools
Source: BMC Med. 2012 Nov 22;10:144. doi: 10.1186/1741-7015-10-144 (PMC3523082; doi:10.1186/1741-7015-10-144)
Supplement: Additional file 3 — App types identified by the systematic assessment. The table shows a numeric breakdown of the types of content and features exposed by apps included in the assessment. [file 1741-7015-10-144-S3.DOC]

**Additional File 3**

App types identified by the systematic assessment

The table shows a numeric breakdown of the types of content and features exposed by apps included in the assessment.

|  | | | | **n (%)** | | **Android** | **Apple** | **Blackberry** | **WP** |
| --- | --- | --- | --- | --- | --- | --- | --- | --- | --- |
|  | | | | | | | | | |
| **All health information apps** | | | | 56 | (100%) | 25 | 26 | 4 | 1 |
|  | | | | | | | | | |
|  | Textual content | | | 43 | (77%) | 20 | 20 | 2 | 1 |
|  |  | eBook | | 12 |  | 8 | 4 | 0 | 0 |
|  |  | Other structured text | | 16 |  | 7 | 7 | 2 | 0 |
|  |  | Flip cards | | 1 |  | 1 | 0 | 0 | 0 |
|  |  | Glossaries and directories | | 14 |  | 4 | 9 | 0 | 1 |
|  |  |  | Directory | 7 |  | 2 | 4 | 0 | 1 |
|  |  |  | Glossary | 6 |  | 1 | 5 | 0 | 0 |
|  |  |  | Link aggregator | 1 |  | 1 | 0 | 0 | 0 |
|  | | | | | | | | | |
|  | Other media | | | 4 |  | 1 | 3 | 0 | 0 |
|  |  | Animation | | 3 | (5%) | 1 | 2 | 0 | 0 |
|  |  | Audio | | 1 | (2%) | 0 | 1 | 0 | 0 |
|  | | | | | | | | | |
|  | Mixed content | | | 9 | (16%) | 4 | 3 | 2 | 0 |
|  | | | | | | | | | |
| **All management tool apps** | | | | 47 | (100%) | 16 | 26 | 4 | 1 |
|  | | | | | | | | | |
|  | Assessment tools^*^ | | | 17 | (36%) | 6 | 10 | 1 | 0 |
|  |  | Questionnaires | | 7 |  | 4 | 3 | 0 | 0 |
|  |  | Predicted peak flow calculators | | 7 |  | 2 | 4 | 1 | 0 |
|  |  | Diagnostic instruments | | 3 |  | 0 | 3 | 0 | 0 |
|  | | | | | | | | | |
|  | Diaries and trackers^*^ | | | 29 | (62%) | 9 | 17 | 2 | 1 |
|  |  | Symptom-only diaries | | 2 |  | 0 | 2 | 0 | 0 |
|  |  | Peak flow-only diaries | | 2 |  | 2 | 0 | 0 | 0 |
|  |  | Medication-only diaries | | 0 |  | 0 | 1 | 0 | 0 |
|  |  | Mixed diaries | | 23 |  | 7 | 13 | 2 | 1 |
|  |  | Medication trackers | | 5 |  | 0 | 4 | 0 | 1 |
|  | | | | | | | | | |
|  | Pollen or pollution alerts^*^ | | | 6 | (11%) | 3 | 3 | 0 | 0 |
|  | | | | | | | | | |
|  | Other tools | | | 2 | (4%) | 0 | 1 | 1 | 0 |
|  | | | | | | | | | |

* Some apps included multiple functions and are therefore counted more than once.
