# Supplementary material for: Apps for asthma self-management: a systematic assessment of content and tools
Source: BMC Med. 2012 Nov 22;10:144. doi: 10.1186/1741-7015-10-144 (PMC3523082; doi:10.1186/1741-7015-10-144)
Supplement: Additional file 5 — Comprehensiveness of educational topic coverage by health information apps. The table summarizes the number of apps addressing each of eight topics defined for asthma self-management education. Each app was assessed as either addressing the domain wholly, partially or not at all, using defined sub-criteria, which are described separately. [file 1741-7015-10-144-S5.DOC]

**Additional File 4**

Comprehensiveness of educational topic coverage by health information apps

The table summarises the number of apps addressing each of 8 topics defined for asthma self-management education. Each app was assessed as either addressing the domain wholly, partially or not at all, using defined sub-criteria which are described separately.

|  | |  | **Number addressing (n=38)** | | |
| --- | --- | --- | --- | --- | --- |
| **Educational topic** | | **%^*^** | **Wholly** | **Partially** | **No** |
| Basic facts about the nature of the condition | | 67% | 8 | 18 | 12 |
| Allergen and trigger avoidance | | 47% | 2 | 16 | 20 |
| The nature of treatment: relievers and preventers | | 45% | 9 | 8 | 21 |
| Recognising and responding appropriately to acute exacerbations | | 36% | 3 | 11 | 24 |
|  | Lay management of acute asthma^†^ | 18% | 0 | 7 | 31 |
| How to use treatment | | 26% | 2 | 8 | 28 |
| Self-monitoring and assessment skills | | 26% | 4 | 6 | 28 |
| The role of a written, personalised action plan | | 18% | 4 | 3 | 31 |
| Personalising the definition of good asthma control | | 8% | 2 | 1 | 35 |

^*^Percentage of apps addressing topic either wholly or partially.

^†^Subcriterion of recognising and responding to acute exacerbations.
