# Supplementary material for: Apps for asthma self-management: a systematic assessment of content and tools
Source: BMC Med. 2012 Nov 22;10:144. doi: 10.1186/1741-7015-10-144 (PMC3523082; doi:10.1186/1741-7015-10-144)
Supplement: Additional file 6 — Consistency of recommendations made by asthma apps with evidence-base. The table shows the number of references to each statement made by apps included in the assessment and the direction of any recommendation associated with that statement. For example, an app claiming that removing pets from the home is beneficial for asthma symptom control would count under the 'Beneficial' column. The expected advice (in the example given: that there is no clear evidence that removal of pets from the home improves asthma symptoms) is shown for each statement in the shaded box. [file 1741-7015-10-144-S6.DOC]

# Additional File 5

Consistency of recommendations made by asthma apps with evidence-base

The table shows the number of references to each statement made by apps included in the assessment and the direction of any recommendation associated with that statement. For example, an app claiming that removing pets from the home is beneficial for asthma symptom control would count under the ‘Beneficial’ column. The expected advice (in the example given: that there is no clear evidence that removal of pets from the home improves asthma symptoms) is indicated for each statement by the column in which the text is emboldened.

| **Statement** | **n** | **Beneficial** | **Uncertain** | **Not beneficial** |
| --- | --- | --- | --- | --- |
| Secondary prophylaxis - removal of pets from the home | 5 | 3 | **2** | 0 |
| Secondary prophylaxis – fungal allergen avoidance and control measures | 9 | 8 | **1** | 0 |
| Secondary prophylaxis - cockroach avoidance and control measures | 7 | 7 | **0** | 0 |
| Secondary prophylaxis - cessation of active smoking | 12 | **12** | 0 | 0 |
| Secondary prophylaxis - avoidance of passive smoking | 13 | **13** | 0 | 0 |
| Secondary prophylaxis - avoidance of exposure to air pollution | 11 | 9 | **2** | 0 |
| Secondary prophylaxis - immunotherapy in atopic asthma | 2 | **2** | 0 | 0 |
| Secondary prophylaxis - weight reduction in obese patients | 7 | **7** | 0 | 0 |
| Secondary prophylaxis - seasonal influenza vaccination | 6 | 5 | **1** | 0 |
